# Rapid Prediction of Adulteration Content in *Atractylodis rhizoma* Based on Data and Image Features Fusions from Near-Infrared Spectroscopy and Hyperspectral Imaging Techniques

**DOI:** 10.3390/foods12152904

**Published:** 2023-07-30

**Authors:** Zhiwei Jiang, Aimin Lv, Lingjiao Zhong, Jingjing Yang, Xiaowei Xu, Yuchan Li, Yuchen Liu, Qiuju Fan, Qingsong Shao, Ailian Zhang

**Affiliations:** 1 State Key Laboratory of Subtropical Silviculture, Zhejiang A&F University, Hangzhou 311300, China13296732737@163.com (Y.L.); sqszjfc@163.com (Q.S.); 2 Zhejiang Provincial Key Laboratory of Resources Protection and Innovation of Traditional Chinese Medicine, Zhejiang A&F University, Hangzhou 311300, China; 3 Wenzhou Forestry Technology Promotion and Wildlife Protection Management Station, Wenzhou 325027, China

**Keywords:** adulteration, *Atractylodes chinensis* (DC.) Koidz., *Atractylodes lancea* (Thunb.) DC., nondestructive analysis, partial least squares regression

## Abstract

*Atractylodis rhizoma* (AR) is an herb and food source with great economic, medicinal, and ecological value. *Atractylodes chinensis* (DC.) Koidz. (AC) and *Atractylodes lancea* (Thunb.) DC. (AL) are its two botanical sources. The commercial fraud of AR adulterated with *Atractylodes japonica* Koidz. ex Kitam (AJ) frequently occurs in pursuit of higher profit. To quickly determine the content of adulteration in AC and AL powder, two spectroscopic techniques, near-infrared spectroscopy (NIRS) and hyperspectral imaging (HSI), were introduced. The partial least squares regression (PLSR) algorithm was selected for predictive modeling of AR adulteration levels. Preprocessing and feature variable extraction were used to optimize the prediction model. Then data and image feature fusions were developed to obtain the best predictive model. The results showed that if only single-spectral techniques were considered, NIRS was more suitable for both tasks than HSI techniques. In addition, by comparing the models built after the data fusion of NIRS and HSI with those built by the single spectrum, we found that the mid-level fusion strategy obtained the best models in both tasks. On this basis, combined with the color-texture features, the prediction ability of the model was further optimized. Among them, for the adulteration level prediction task of AC, the best strategy was combining MLF data (at CARS level) and color-texture features (C-TF), at which time the R^2^_T_, RMSET, R^2^_P_, and RMSEP were 99.85%, 1.25%, 98.61%, and 5.06%, respectively. For AL, the best approach was combining MLF data (at SPA level) and C-TF, with the highest R^2^_T_ (99.92%) and R^2^_P_ (99.00%), as well as the lowest RMSET (1.16%) and RMSEP (2.16%). Therefore, combining data and image features from NIRS and HSI is a potential strategy to predict the adulteration content quickly, non-destructively, and accurately.

## 1. Introduction

*Atractylodis rhizoma* (AR) is the rhizome of the famous perennial herb *Atractylodes lancea* (Thunb.) DC. (AL) or *Atractylodes chinensis* (DC.) Koidz. (AC), which has high economic, medicinal, and ecological value. Wild AR plants grow mainly on mountain slopes in grasslands, forest understories, thickets, and rock crevices. Nowadays, AR is widely cultivated in East and Southeast Asian countries such as China, Japan, Thailand, and Korea. Besides its distinctive aroma and capacity to treat digestive, respiratory, and neurological system illnesses, AR is frequently employed as a spice and flavoring agent in the food industry [1,2,3,4]. Therefore, it is regarded as an important cash crop.

Due to the destruction and overexploitation of its natural habitat, AR is facing a scarcity of wild resources, leading to an increase in its economic value and triggering a series of illegal practices of AR fraud in the market. *Atractylodes japonica* Koidz. ex Kitam. (AJ) is one of its common counterfeits, which is primarily cultivated in the northeastern provinces of China and sold as AR despite not being listed in the Chinese Pharmacopoeia. AJ and AR have different chemical compositions, prices, and efficacy concerns [5]. Without the right detection technology to curb this fraud, it will inevitably cause confusion in market management and affect the quality and efficacy of AR.

Conventional identification methods such as thin-layer chromatography, high-performance liquid chromatography, and DNA molecular labeling techniques may be able to determine whether AR is adulterated, but they have the disadvantage of being time-consuming, cumbersome, and operator-demanding [6,7,8]. Therefore, in recent years, the search for rapid non-destructive testing techniques has become more vocal. Near-infrared spectroscopy (NIRS) and hyperspectral imaging (HSI) techniques are typical of rapid non-destructive testing techniques, and they are widely used in the fields of food and medicine. NIRS combined with chemometrics can predict the adulteration level of ginger powder, *Ganoderma lucidum* spore powder, and honeysuckle [9,10,11]. And HSI combined with chemometrics has also been reported to successfully predict the adulteration concentration of oregano, turmeric powder, and saffron [12,13,14]. Additionally, the concepts of data fusion and image feature fusion have been proposed in the fields of food and medicine. Some studies have indicated that data fusion or combining image features can identify the origin and variety of rice, predict the polyphenol content and antioxidant capacity of peppercorns, and detect the soluble solids content in red globe grapes [15,16,17]. The application of NIRS combined with chemometrics has been reported to successfully identify the authenticity, botanical origin, and geographical origin of AR decoction pieces, as well as predict the atractylodin content of bran-fried AR [18,19]. However, there has been no study on the use of NIRS and HSI to predict the adulteration content in AR.

In previous studies, research has typically focused on a single spectral technique, and image features are generally combined with HSI data. In this study, the focus has been extended to data fusion using two spectral techniques and adding image features based on them. Data fusion provides a method to combine NIRS and HSI spectral data based on complementary or synergistic effects, which can usually be divided into low-level fusion (LLF), mid-level fusion (MLF), and high-level fusion (HLF) strategies depending on the data structure [20]. Because HLF is susceptible to severe information loss when forecasting model performance, LLF and MLF were taken into consideration in this work [21]. HSI offers the possibility of extracting image features in addition to spectral data. Color and texture features are generally valuable image features, which has also been confirmed in previous studies [22]. In the present study, based on the superior performance of the data and image feature fusions of NIRS and HSI in previous studies, we speculated that these may also have the potential to be applied to the prediction of adulteration content in AR. To test this hypothesis, we mainly performed the following work: (i) establishing a partial least squares regression (PLSR) prediction model for the adulteration concentration of AC and AL powder doped with AJ; (ii) optimizing the model using pretreatment (SGS, SNV, MSC, 1 Der and 2 Der) and feature variable extraction (CARS, SPA, and GA); (iii) introducing a data and image feature fusion strategy to make the model performance further optimized. Finally, this study establishes a new method for rapid nondestructive detection of the amount of AJ doped in AR, which provides new ideas for the quality evaluation of herbs and food and a theoretical basis for the improvement of the quality evaluation system of AR and is conducive to the benign development of Chinese herbal medicine.

## 2. Materials and Methods

### 2.1. Sample Collection and Processing

AC decoction pieces were mainly collected from China province: Anhui (1), Gansu (3), Guangxi (1), Hebei (1), Heilongjiang (5), Jilin (3), Liaoning (4), Inner Mongolia (12), Shaanxi (1), Sichuan (2), Yunnan (1), and Zhejiang (1). AL decoction pieces were collected from Anhui (7), Guangxi (1), Henan (1), Hubei (8), Jiangsu (3), Jiangxi (1), Shaanxi (2), and Zhejiang (1). AJ decoction pieces were collected from Liaoning. All samples were cleaned of impurities, dried, numbered, and powdered, and passed through a 50-mesh sieve in accordance with the adulteration content of 0, 10%, 20%, 30%, 40%, 50%, 70%, and 90% to make a total weight of 2 g. For each level, one sample was prepared, and a sum of 35 pure and 245 adulterated AC powders and 24 pure and 168 adulterated AL powders were obtained.

### 2.2. Spectral Acquisition and Image Feature Extraction

All samples were scanned by the NIR spectrometer (Antaris™ II, Thermo Fisher Scientific Co., Ltd., Waltham, USA) and hyperspectral imager (GaiaField-N17E, Sichuan Shuangli Hepu Technology Co., Ltd., Chengdu, China). The parameters of the NIR spectrometer were set as follows: the number of spectral scans was 32, and the resolution was 8 cm^−1^. The parameters of the hyperspectral imager were set as follows: The moving speed and distance of the carrier platform were 1.4 cm s^-1^ and 30 cm, respectively; the vertical distance between the sample and the lens was 42 cm; and the exposure time was 7 ms. For NIRS, the scan was repeated three times for each sample and averaged for subsequent data analysis. As for HSI, it was scanned once, as in previous studies [21]. Finally, 1557 NIRS data from 4000–10,000 cm^−1^ and 512 HSI data from 900–1700 nm were acquired.

**Figure 1:**
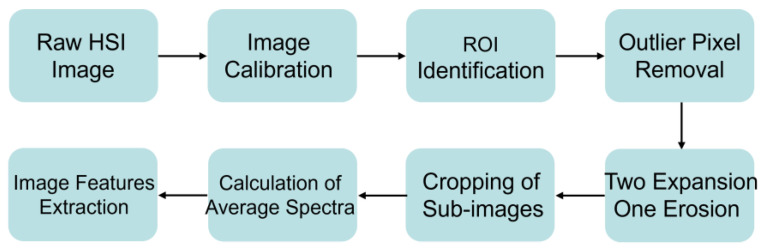
Schematic diagram of hyperspectral data and the image feature extraction process.

The raw NIRS data was entered into the Unscrambler X 10.4 software to extract the corresponding spectral values. As shown in Figure 1, before HSI data could be used for further analysis, the following four processes had to be completed: (i) The original hyperspectral image was calibrated to create a calibrated hyperspectral image using Formula (1); (ii) The calibrated hyperspectral image was input and applied to the mask image to identify the region of interest (ROI) and remove outlier pixels; (iii) Two expansion and one erosion operations were performed; (iv) Each image was cropped into sub-images and averaged the reflectance values of all pixels in the sub-images to obtain the average spectrum for each sample. In addition, the study also considered the combination of color and texture features to improve the prediction ability. Color features (CF) were selected from first-order moments, second-order moments, and third-order moments of the three channels of RGB, while texture features (TF) included energy, homogeneity, contrast, and the correlation of each pixel in the Gray-Level Co-occurrence Matrix (GLCM) at three bands (47, 197, 378) and four directions (0°, 45°, 90°, 135°). Masked and filtered binary images were used to calculate the morphological features. A total of 48 texture features were extracted from the new hypercube by the GLCM algorithm, and 9 color features were calculated from the three RGB channels (Table S1) [23,24].

(1) $$RC=\frac{RO-RB}{RW-RB}$$

Note: *RB* = the all-black background image; *RC* = the corrected image; *RO* = the original image; *RW* = the all-white calibration plate image.

### 2.3. Preprocessing and Feature Variable Extraction

To remove systematic noise and outside environmental influences during sample collection, as well as to reduce the substantial amount of redundant and useless spectral data information in the full wavelength, simplify the model, and enhance its predictive power, pretreatment and feature variable extraction were required [25]. As previous studies, five pretreatment methods such as Savitzky-Golay smoothing (SGS), standard normalized variate (SNV), multiplicative scatter correction (MSC), the first derivative (1 Der), and the second derivative (2 Der) were chosen. Among them, SGS is used for smoothing filtering, which reduces the interference of noise on the sampled signal. The inhomogeneity of the sample causes light scattering, which leads to errors in the sample spectrum, and SNV can remove additive and multiplicative effects from the spectrum. MSC is mainly applied to eliminate scattering effects caused by inhomogeneity in particle distribution and particle size. Interference caused by baseline drift or soft background can be eliminated by 1 Der and 2 Der algorithms, as can overlapping peaks, to improve resolution and sensitivity [26].

In addition, three feature variable extraction methods, such as competitive adaptive reweighted sampling (CARS), successive projection algorithms (SPA), and genetic algorithms (GA), were selected [15]. CARS is an efficient wavelength selection algorithm based on the principle of “survival of the fittest.” CARS is designed to select critical wavelengths through a rigorous and computationally efficient procedure. Two consecutive wavelength selection steps are performed: in the first step, wavelengths with relatively small PLSR coefficients are forcibly removed using an exponential decay function. Next, variable sampling with adaptive reweighting is used to further eliminate wavelengths in a competitive manner. SPA is a forward variable selection algorithm used for multivariate calibration to select wavelengths with minimal redundancy. It performs simple projection operations in vector space to obtain a subset of useful variables with minimal covariance. The principle of variable selection via SPA is that the newly selected variable is the one with the largest projection on the orthogonal subspace of the previously selected variable. The optimal initial variables and the number of variables can be determined based on the minimum root-mean-square error of cross-validation. GA is a global optimization search method inspired by Darwin’s theory of natural selection. It selects variables that are better suited to the fitness function through the manipulation of genetic processes, such as reproduction, mutation, and selection, and successive genetic iterations. In this study, the parameter values of the GA were set based on preliminary tests: population size (30), window width (3), penalty slope (0), maximum generations (100), mutation rate (0.01), crossover probability (0.5), and replicate runs (100) [27].

### 2.4. Data and Image Feature Fusions

**Figure 2:**
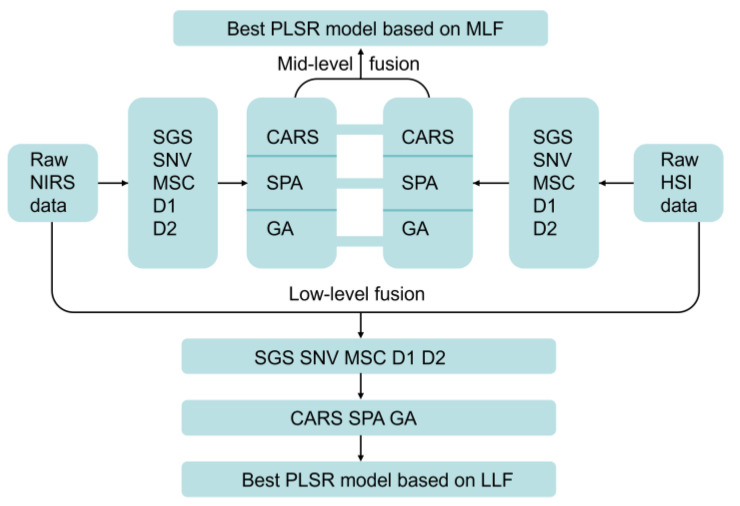
Schematic diagram of data fusion strategy.

The NIRS and HSI data are directly stitched together to obtain the LLF dataset, and the LLF is usually used in conjunction with the feature variable extraction method because more information is introduced to interpret the sample. The features extracted by the same feature filtering method are integrated into a new MLF dataset, which effectively avoids the drawbacks of LLF by integrating data from two sources without causing a large increase in data [28]. NIRS, HSI, and LLF would go through five pretreatments and three feature variable selections to filter out the best combination of their respective methods, whereas MLF built an optimal PLSR model by fusing the NIRS and HSI feature variables extracted by the same feature variable extraction methods based on the best pretreatment (Figure 2). CF, TF, or color-texture features (C-TF) would be added to the best PLSR models based on NIRS, HSI, LLF, or MLF to obtain the optimal model.

### 2.5. Data Set Partitioning and Quantitative Analysis Methods

The joint x-y distance (SPXY) algorithm uses a partitioning algorithm that accepts the variability of x-space and y-space. In contrast to partitioning systems based just on x information or random sampling, the multidimensional space can be covered more effectively with this approach [29]. Therefore, SPXY was proposed to divide the dataset. The dataset was divided into training and prediction sets in the ratio of 4:1. For AC doped concentration prediction, the numbers of samples in the training and prediction sets were 672 and 168, respectively, while for AL, the numbers were 461 and 115, respectively. PLSR is a common modeling method for quantitative spectral analysis that effectively solves the issue of multiplicity correlation between variables and incorporates the principles and characteristics of principal component analysis, multiple linear regression, and conventional correlation analysis. Because of its forceful predictive power, the PLSR algorithm was chosen as the quantitative prediction model [30,31]. The choice of principal component number affects the modeling effect of PLSR; therefore, 10-fold cross-validation was proposed to select the optimal principal component number to minimize the root mean square error (RMSE). In this study, the performance of the model was assessed mainly by the correlation coefficient of training sets (R^2^_T_), the correlation coefficient of prediction sets (R^2^_P_), the root mean square error of training sets (RMSET), and the root mean square error of prediction sets (RMSEP). A good model ought to have a low RMSE and a high R^2^. In addition, the principal component number should be as small as possible, because a large principal component number may introduce some irrelevant information and cause overfitting of the model [32].

### 2.6. Software

The Unscrambler X 10.4 was used for NIRS data format conversion, and SpecView was used for hyperspectral image capture and calibration. The rest of the data processing was conducted on MATLAB R2022a.

## 3. Results and Discussion

### 3.1. Sample and Spectral Analysis

**Figure 3:**
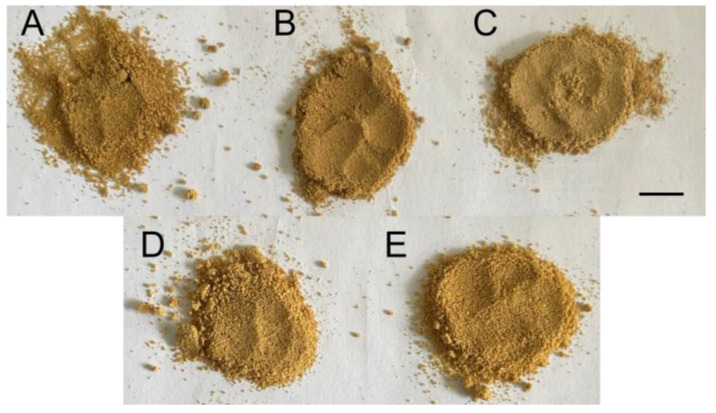
Samples of AR. (**A**) pure AC powder; (**B**) adulterated AC powder (50% adulteration content); (**C**) AJ powder; (**D**) pure AL powder; (**E**) adulterated AL powder (50% adulteration content). Scale bar = 1 cm.

**Figure 4:**
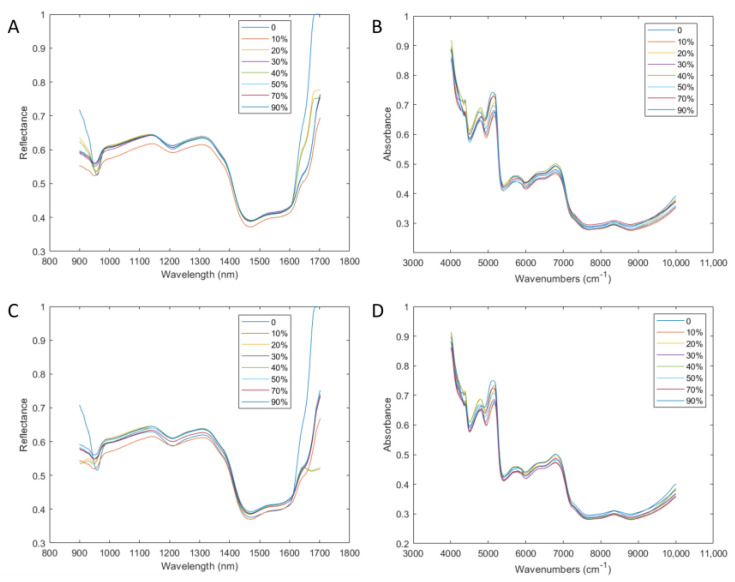
HSI ((**A**): AC; (**C**): AL) and NIRS ((**B**): AC; (**D**): AL) spectrograms of pure and adulterated AC and AL (adulteration concentrations: 0, 10%, 20%, 30%, 40%, 50%, 70%, 90%). Note: AC from Anhui, Gansu, Guangxi, Hebei, Heilongjiang, Jilin, Liaoning, Inner Mongolia, Shaanxi, Sichuan, Yunnan, and Zhejiang; AL from Anhui, Guangxi, Henan, Hubei, Jiangsu, Jiangxi, Shaanxi, and Zhejiang.

The color, texture, and size of the powder particles were relatively similar between pure and adulterated AR powder, so it was challenging to quickly identify them from their appearance (Figure 3). Unsurprisingly, the raw spectrograms did not seem to work well either. The curve trends of pure and adulterated samples were similar, and the peaks appeared at the same position and height, whether in the HSI or NIRS spectrogram (Figure 4), which was consistent with previous studies [33,34]. Therefore, it was necessary to perform specific processing on the raw spectra to achieve a fast and accurate prediction of the adulteration level.

### 3.2. Quantitative Analysis Based on NIRS Data and Image Features

**Figure 5:**
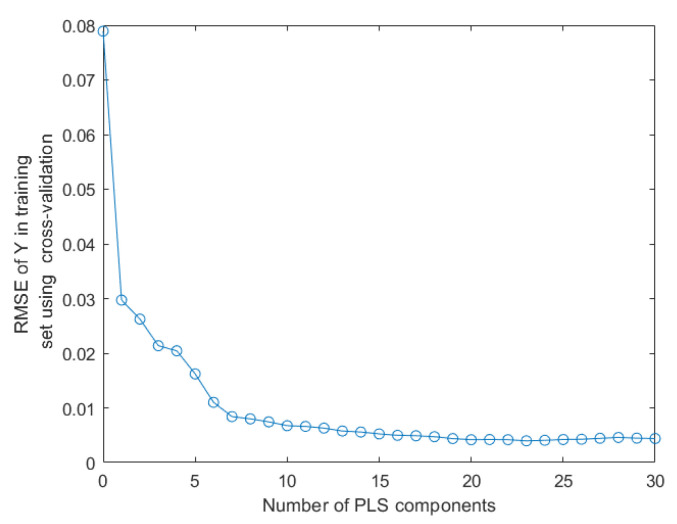
Plot of the RMSE of Y with principal components. Note: There was the SGS + PLSR model for adulterated AC based on NIRS data.

For predicting the adulteration concentration of AC samples, the PLSR model based on the original data obtained high R^2^_T_ (95.32%) and R^2^_P_ (95.00%) and acceptable RSMET (8.63%) and RMSEP (8.89%) (Table 1). All five preprocessing methods improved the model’s performance to different degrees. Among them, 1 Der obtained the best result since the model had higher R^2^_T_ (99.77%) and R^2^_P_ (97.61%) as well as lower RMSET (1.94%) and RMSEP (6.60%). 2 Der was not considered because its R^2^_P_ (87.45%) and RMSEP (15.53%) were suboptimal, even though its R^2^_T_ (99.98%) and RMSET (0.60%) were excellent, while the feature variable extraction degraded the model’s performance to different degrees. Although CARS only degraded the model slightly, the modeling efficiency increased substantially; hence, CARS was chosen as the best feature variable extraction method, at which time the model’s R^2^_T_, R^2^_P_, RMSET, and RMSEP were 99.06%, 3.87%, 98.50%, and 5.14%, respectively. The principal component score selection was obtained by plotting the RMSE of Y with the principal components. In Figure 5, the smallest RMSE was obtained by cross-validation when the number of principal components was 20 (when the number of principal components was between 10 and 20, the RMSE value showed a downward trend and was basically stable after 20, so 20 was selected). Since previous studies have proved that HSI and color feature fusion had good modeling efficacy for whole wheat flour samples with different DON levels and that introducing a large number of features without reducing the data dimension may complicate the model, CF, TF, and C-TF were added after the feature variables were extracted [34,35]. It was obvious that the best performance of the model was obtained by combining CT. At this time, R^2^_T_, RMSET, R^2^_P_, and RMSEP were 99.06%, 3.87%, 98.50%, and 5.14%, respectively. Only 85 features were combined; compared with the original data, the features were reduced by 94.54%, while R^2^_T_ and R^2^_P_ were improved by 3.74% and 3.50%, respectively, and RMSET and RMSEP were reduced by 4.76% and 3.75%, respectively.

For predicting the adulteration level of AL, the PLSR model built from the raw NIRS data had R^2^_T_, RMSET, R^2^_P_, and RMSEP of 97.81%, 5.93%, 92.45%, and 11.73%, respectively (Table 1). The preprocessing did not optimize the model in all cases, especially with 2 Der, where the R^2^_P_ was reduced by 24.29% and the RMSEP increased by 9.36%. While previous studies found significant performance gains in models built by choosing 2 Der as preprocessing, our study reached the opposite results [9]. A reasonable explanation was that the signal-to-noise ratio decreases as the derivative increases, and the spectral information may be lost [36]. The best pretreatment choice was SGS, where the R^2^_T_, RMSET, R^2^_P_, and RMSEP were 99.66%, 2.24%, 97.60%, and 7.08%, respectively. The best feature selection method was SPA, where 95 feature variables were filtered, a reduction of 1462 compared to the full wavelength. However, neither feature variable selection nor combining image features led to improved model performance, presumably because some of the useful information was eliminated and the algorithm utilized different information with different efficiency [37].

### 3.3. Quantitative Analysis Based on HSI Data and Image Features

The models based on HSI data performed worse than those based on NIRS. For predicting the adulteration content of the adulterated AC, the R^2^_T_, RMSET, R^2^_P_, and RMSEP of the model using the original data were only 81.92%, 16.39%, 79.99%, and 17.24%, and after preprocessing with 2 Der, the R^2^ could exceed 88% (Table 1). The best feature variable extraction method was SPA, at which time the R^2^_T_ could reach 90.06%. Although R^2^_P_ was only 85.59%, it was still the highest among the three methods. We tried to combine image features on the basis of 2 Der + SPA and found that the model results combining three image features were in the order of good to bad: 2 Der + SPA + CF > 2 Der + SPA + T-CF > 2 Der + SPA + TF. Similar results were found in the classification task [38], and it might be that the TF carries some useful information and the others may not contribute much to the modeling. Although the R^2^ of the model constructed by 2 Der + SPA + CF was higher than 86%, it was still far from the best PLSR model based on NIRS data.

Interestingly, in predicting the adulteration level in AL, the model built with original data gave better results than that built with pretreatments, except for 2 Der. After 2 Der processing, the R^2^_T_, RMSET, R^2^_P_, and RMSEP of the model were 99.12%, 3.69%, 91.66%, and 11.58%, respectively, which changed by 9.46%, 8.56%, 2.89%, and 4.03% compared to the original data (Table 1). In addition, we found that 2 Der was the best choice for predicting the adulteration level of either AC or AL based on HSI data. In the detection of metanil yellow adulteration in chickpea flour based on the HSI full spectrum, the best pretreatment was also 2 Der, and our findings were consistent with this [33]. This might be related to the ability of 2 Der to separate overlapping peaks, which may lead to the success prediction [39]. The best feature variable selection method was then SPA, at which point it filtered to 94 feature wavelengths. Combining TF on the basis of this seemed to be the best option. However, in general, feature variable extraction and combining TF did not lead to a significant improvement in model performance. Furthermore, the model built from HSI data seemed to be worse than the one built from NIRS, which was explainable because HSI shows its unique spatial resolution at the expense of spectral resolution compared to conventional NIRS spectroscopy [40]. However, when NIRS is combined with the C-TF method extracted from HSI, the complementary advantages of the two enable the model performance to be further optimized, which gave us the possibility to think about whether a better model can be obtained by fusing the two data and then combining the image features.

### 3.4. Quantitative Analysis Based on LLF Data and Image Features

The LLF dataset of 2069 features was obtained by integrating 1557 variables in the NIRS and 512 variables in the HSI. For the adulteration content prediction of adulterated AC, 1 Der was the best pretreatment choice, at which time R^2^_T_, RMSET, R^2^_P_, and RMSEP were 99.92%, 1.15%, 97.71%, and 6.73%, respectively (Table 1). 117 feature variables were obtained after SPA extraction of feature variables, which was 94.35% less compared to total variables, but there was a slight decrease in the performance of the model. There was further degradation in the performance of the model if image features were introduced.

For predicting the adulteration level of AL, its combination with the original data could obtain 98.16%, 5.47%, 91.95%, and 12.29% for R^2^_T_, RMSET, R^2^_P_, and RMSEP (Table 1). After pretreatment, SGS, SNV, MSC, and 1 Der could optimize the model, while 2 Der did the opposite. Among them, the model performed best after SGS treatment compared with the original data, with R^2^_T_ and R^2^_P_ increasing by 1.65% and 5.24%, respectively, while RMSET and RMSEP decreasing by 3.72% and 5.92%, respectively. However, similar to the AC prediction task, both feature variable selection and combining image features made the modeling worse. LLF did not seem to be suitable for combining image features to accomplish the above two prediction tasks. In addition, the models using LLF exhibited similar or poorer performance compared to models built with a single spectrum. However, in the previous study, the LLF strategy had better predictive ability in predicting TVB-N content in chicken than the optimal model for single-spectral data. Our results yielded different conclusions from this, which could be attributed to the large amount of data introduced by LLF, leading to the computational complexity and uncertainty of the PLSR method [21,41].

### 3.5. Quantitative Analysis Based on MLF Data and Image Features

Based on the best pretreatment, the MLF dataset was obtained by integrating the feature variables obtained by the same feature variable extraction method. For predicting the adulteration content of AC, the model built at the CARS level was the best, with R^2^_T_, RMSET, R^2^_P_, and RMSEP of 99.15%, 3.61%, 98.17%, and 6.55%, respectively (Table 1). If image features were considered, C-TF was the best choice because the R^2^_T_, RMSET, R^2^_P_, and RMSEP were 99.85%, 1.25%, 98.61%, and 5.06%, respectively, which was superior to the best model built based on NIRS data.

For predicting the adulteration content in AL, the model built at the SPA level was the best, with R^2^_T_, RMSET, R^2^_P_, and RMSEP of 99.62%, 2.37%, 96.22%, and 11.00%, respectively (Table 1). Similarly, the combination of C-TF on this basis also achieved the best results, with R^2^_T_, RMSET, R^2^_P_, and RMSEP reaching 99.92%, 1.16%, 99.00%, and 2.16%, better than the best models built with single spectral or LLF data. In general, the MLF strategy achieved very good results, which was consistent with the findings of previous studies [15].

### 3.6. Analysis of Feature Variables

**Figure 6:**
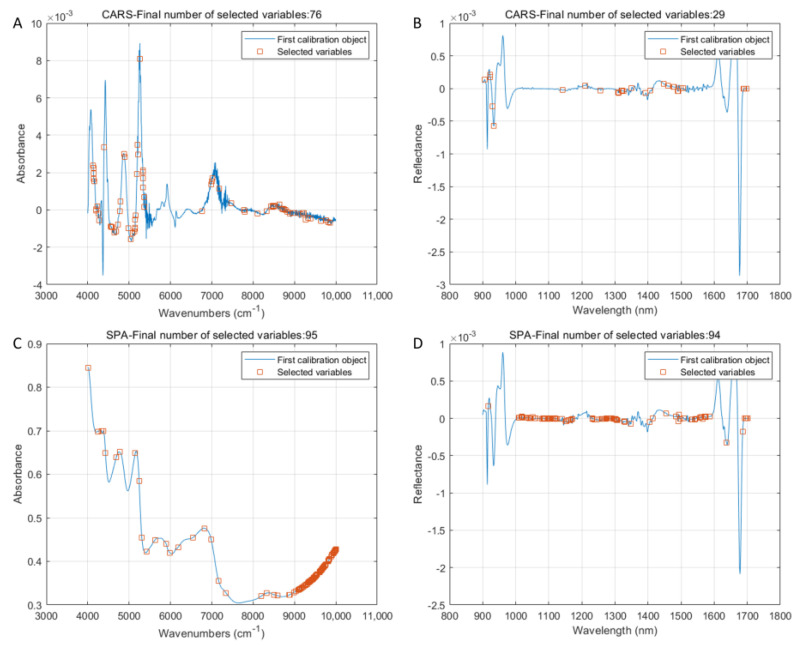
Feature variables selected by the MLF strategy. Note: (**A**,**B**) denote the NIRS (1 Der + CARS) and HSI datasets (2 Der + CARS) used to predict the adulteration level of adulterated AC powder, respectively. (**C**,**D**) denote the NIRS (SGS + SPA) and HSI datasets (2 Der + SPA) used to predict the adulteration level of adulterated AL powder, respectively.

The characteristic variables selected for the MLF strategy were analyzed to find the reasons for the success of the adulterated content prediction. In Figure 6A, the feature variables were mainly distributed in the ranges of 4200–5300 cm^−1^ and 8200–9800 cm^−1^. Unfortunately, there is little information in the scientific literature about the NIRS of AR. However, an attempt would be made to identify the compounds that may be present in the NIRS of AR. We speculated that the feature variables selected between 4200 and 5300 cm^−1^ may be related to the absorption valleys near 4200, 4600, and 5000 cm^−1^. The variables near 4600 and 5000 cm^−1^ may be connected to the combined C-H and N-H bonding vibrations found in proteins. Those near 4200 cm^−1^ could be attributed to the C-H and C-C bonding vibrations [42]. The region between 8200 and 9800 cm^−1^ was associated with the second overtone vibrations of C-H and N-H [43]. In Figure 6B, the characteristic variables were mainly distributed between 1140 and 1500 nm, which may be related to the first-order octave vibrations of C-H and N-H.

Intriguingly, the feature variables in Figure 6C were primarily dispersed between the ranges of 5200–7300 and 8200–9800 cm^−1^. The absorption band of 5200–7300 cm^−1^ might be due to the amide combination band vibration of CONH_2_ and the N-H stretching of the protein [44]. When it came to feature variables of HSI data processed by 2 Der and SPA (Figure 6D), selected feature variables concentrated in 1000–1180, 1220–1340, and 1450–1600 nm could be observed. Feature variables in the 1000–1180 nm range may be influenced by the second overtone of N-H or O-H [36]. In summary, proteins may affect the effectiveness of model modeling, and the specific substances still need further in-depth study.

## 4. Conclusions

To predict the adulteration content of AC and AL powder quickly and nondestructively, two spectroscopic techniques, NIRS and HSI, were developed. Preprocessing, feature variable selection, data fusion, and image feature fusion strategies were introduced to obtain the optimal prediction model. The results suggested that a single NIRS might be more appropriate than a single HSI, but if higher prediction results were pursued, adding C-TF data to the MLF would be a better choice. To be specific, for the task of predicting the adulteration level of AC, the best strategy was combining MLF data (at CARS level) and C-TF, at which time the R^2^_T_, RMSET, R^2^_P_, and RMSEP of the model were 99.85%, 1.25%, 98.61%, and 5.06%, respectively. For another task, the best approach was adding C-TF to the basic MLF data (at SPA level), where the R^2^_T_, RMSET, R^2^_P_, and RMSEP were 99.92%, 1.16%, 99.00%, and 2.16%, respectively. In addition, we found that proteins may be one of the factors affecting successful modeling, but the exact substance still needs further study. The instruments used in this study are suitable for analytical testing in the laboratory; therefore, the use of low-cost and convenient spectroscopic equipment for quality assessment in the field or considering applying a demixing algorithm to obtain a better model may be the focus of future work. In conclusion, the data and image feature fusions based on NIRS and HSI can predict the level of adulteration of AR powder quickly, nondestructively, and accurately, which is beneficial to safeguarding the quality and efficacy of herbs as well as providing a theoretical basis and new ideas for quality evaluation of Chinese herbs. 

## Figures and Tables

**Table 1:** PLSR Modeling results under different methods of processing.

Object	Data	Method	Principal Components Number	Variables Number	R^2^_T_ (%)	RMSET (%)	R^2^_P_ (%)	RMSEP (%)
Adulterated AC	NIRS	Raw data	21	1557	95.32	8.63	95.00	8.89
		SGS	20	1557	97.21	6.79	96.82	6.83
		SNV	21	1557	97.72	6.20	95.88	7.51
		MSC	20	1557	97.76	5.82	96.90	7.73
		1 Der	15	1557	99.77	1.94	97.61	6.60
		2 Der	9	1557	99.98	0.60	87.45	15.53
		1 Der + CARS	10	76	99.04	3.91	97.34	6.88
		1 Der + SPA	4	74	93.96	9.93	86.49	14.25
		1 Der + GA	7	24	95.87	8.45	90.33	9.90
		1 Der + CARS + CF	15	85	99.06	3.87	98.50	5.14
		1 Der + CARS + TF	32	124	98.99	4.09	98.07	5.31
		1 Der + CARS + C-TF	42	133	98.96	4.14	97.77	5.81
	HSI	Raw data	22	512	81.92	16.39	79.99	17.24
		SGS	21	512	81.68	16.39	81.42	17.15
		SNV	21	512	83.71	15.30	77.17	19.58
		MSC	20	512	83.69	15.70	81.27	16.47
		1 Der	12	512	86.90	14.31	84.44	14.62
		2 Der	9	512	88.25	12.97	88.35	15.39
		2 Der + CARS	5	29	85.66	14.32	85.06	15.78
		2 Der + SPA	10	76	89.99	12.65	85.59	15.16
		2 Der + GA	3	10	84.59	14.93	80.86	18.00
		2 Der + SPA + CF	19	85	93.46	9.58	90.48	14.59
		2 Der + SPA + TF	15	124	88.13	13.39	81.30	17.62
		2 Der + SPA + C-TF	11	133	90.39	11.83	86.13	16.38
	LLF	Raw data	24	2069	96.00	7.89	95.55	8.80
		SGS	27	2069	97.36	6.50	96.58	7.52
		SNV	21	2069	98.53	4.90	97.14	7.36
		MSC	29	2069	98.70	4.60	95.19	8.86
		1 Der	27	2069	99.92	1.15	97.71	6.73
		2 Der	15	2069	99.72	2.14	91.63	11.58
		1 Der + CARS	10	22	96.54	7.61	94.87	8.12
		1 Der + SPA	24	117	99.12	5.56	96.37	7.91
		1 Der + GA	15	75	98.02	5.63	94.03	10.73
		1 Der + SPA + CF	26	126	98.52	4.93	96.16	8.07
		1 Der + SPA +TF	35	165	98.26	5.56	95.07	8.85
		1 Der + SPA + C-TF	34	174	98.31	5.20	97.31	6.95
	MLF	CARS	10	105	99.15	3.61	98.17	6.55
		SPA	9	150	96.44	7.61	86.24	15.81
		GA	10	34	96.58	7.50	95.40	8.30
		CARS + CF	16	114	99.09	3.75	98.53	5.28
		CARS + TF	39	153	99.22	3.56	98.03	5.58
		CARS + C-TF	41	162	99.85	1.25	98.61	5.06
Adulterated AL	NIRS	Raw data	21	1557	97.81	5.93	92.45	11.73
		SGS	30	1557	99.66	2.24	97.60	7.08
		SNV	29	1557	99.87	1.43	96.37	8.47
		MSC	27	1557	99.92	1.10	92.76	11.78
		1 Der	11	1557	98.67	4.74	82.63	14.20
		2 Der	10	1557	99.90	1.26	68.16	21.09
		SGS + CARS	9	14	91.69	11.19	86.63	15.42
		SGS + SPA	10	95	98.74	4.39	96.06	10.35
		SGS + GA	7	63	97.02	6.76	93.19	12.40
		SGS + SPA + CF	11	104	99.33	3.23	95.03	10.05
		SGS + SPA + TF	10	143	96.19	7.53	88.57	15.05
		SGS + SPA + C-TF	13	152	99.65	2.32	92.16	12.14
	HSI	Raw data	21	512	89.66	12.25	88.77	15.61
		SGS	23	512	91.79	11.43	79.02	17.12
		SNV	21	512	94.34	9.43	87.54	14.25
		MSC	22	512	91.11	11.83	84.70	14.98
		1 Der	9	512	93.43	10.11	87.15	14.23
		2 Der	9	512	99.12	3.69	91.66	11.58
		2 Der + CARS	6	35	93.10	10.39	87.09	14.48
		2 Der + SPA	8	94	98.01	5.60	88.73	13.97
		2 Der + GA	4	16	86.67	13.52	85.03	17.71
		2 Der + SPA + CF	10	103	99.66	2.38	83.00	17.21
		2 Der + SPA + TF	9	142	99.66	2.26	86.52	16.78
		2 Der + SPA + C-TF	9	151	86.32	14.54	76.61	17.66
	LLF	Raw data	36	2069	98.16	5.47	91.95	12.29
		SGS	40	2069	99.81	1.75	97.19	6.37
		SNV	33	2069	99.92	1.10	95.55	10.20
		MSC	44	2069	99.91	1.19	95.80	10.56
		1 Der	11	2069	99.49	2.90	92.68	10.78
		2 Der	8	2069	99.14	3.72	88.86	13.27
		SGS + CARS	13	44	93.27	10.31	86.60	14.98
		SGS + SPA	30	110	99.05	3.86	92.82	12.16
		SGS + GA	15	116	98.50	4.83	92.27	13.10
		SGS + SPA + CF	24	119	99.30	3.42	90.24	12.05
		SGS + SPA + TF	19	158	99.51	2.79	90.94	17.42
		SGS + SPA + C-TF	20	167	98.69	4.47	86.25	16.38
	MLF	CARS	15	49	95.80	8.11	94.34	10.07
		SPA	23	189	99.62	2.37	96.22	11.00
		GA	20	79	97.95	5.54	95.13	11.17
		SPA + CF	23	198	99.62	2.37	98.53	5.28
		SPA + TF	28	237	99.76	2.02	97.87	5.85
		SPA + C-TF	34	246	99.92	1.16	99.00	2.16

Note: AC = *Atractylodes chinensis* (DC.) Koidz.; AL = *Atractylodes lancea* (Thunb.) DC.; NIRS = near-infrared spectroscopy; HIS = hyperspectral imaging; SGS = Savitzky-Golay smoothing; SNV = standard normalized variate; MSC = multiplicative scatter correction; 1 Der = first derivative; 2 Der = second derivative; CARS = competitive adaptive reweighted sampling; SPA = successive projection algorithm; GA = genetic algorithm; CF = color features; TF = texture features; C-TF = color-texture features; R^2^_T_ = correlation coefficient of training sets; RMSET = root mean square error of training sets; R^2^_P_ = correlation coefficient of prediction sets; RMSEP = root mean square error of prediction sets.

## Data Availability

The data in this study were available from the following sources: the corresponding authors. These data are not publicly available due to the requirement to fund research projects.

## Supplementary Materials

The following supporting information can be downloaded at: https://www.mdpi.com/article/10.3390/foods12152904/s1.
